# Apoptosis induced by the Tibetan herbal remedy PADMA 28 in the T cell-derived lymphocytic leukaemia cell line CEM-C7H2

**DOI:** 10.1186/1477-3163-4-15

**Published:** 2005-09-02

**Authors:** Marcel Jenny, Wolfgang Schwaiger, David Bernhard, Oliver A Wrulich, Daria Cosaceanu, Dietmar Fuchs, Florian Ueberall

**Affiliations:** 1 Division of Medical Biochemistry, Biocenter, Innsbruck Medical School, Fritz Pregl-str. 3, 6020, Innsbruck, Austria; 2 Division of Experimental Pathophysiology and Immunology, Biocenter, Innsbruck Medical School, Fritz Pregl-str.3, 6020, Innsbruck, Austria; 3 Department of Oncology-Pathology, Karolinska Institute, Cancer Centrum R 8:00, 17176, Stockholm, Sweden; 4 Division of Biological Chemistry, Biocenter, Innsbruck Medical School, Fritz Pregl-Str. 3, 6020, Innsbruck, Austria.

**Keywords:** Apoptosis, Bcl-2, T cell-derived lymphocytic leukaemia cells, Multicomponent therapeutics, Tibetan medicine, PADMA 28

## Abstract

The Tibetan herbal remedy PADMA 28 revealed promising results to support treatment of atherosclerosis, Charot syndrome (intermittent claudication), chronic active hepatitis and infection of the respiratory tract. The remedy was confirmed to be closely linked with anti- and pro-oxidative properties *in vitro*. In this study, apoptogenic and survival effects of PADMA 28 were investigated in the T cell-derived lymphocytic leukaemia cell line CEM-C7H2.

PADMA 28 led to a concentration-dependent inhibition of cell proliferation accompanied by the accumulation of CEM-C7H2 cells in subG1 phase, fragmentation of poly (ADP-ribose) polymerase (PARP) and nuclear body formation. Treatment with PADMA 28 rescued to some extent cells over-expressing Bcl-2 from apoptosis. This finding suggests that the mechanism of action of PADMA 28 may be via interference with Bcl-2 triggered survival pathways.

## Background

In its composition, PADMA 28 largely corresponds to an herbal formula derived from Tibetan medicine that was used in Poland in the first half of the 20^th ^century against chronic inflammatory diseases. In the past two decades, this formula has been administered with or without boiled water cooled to lukewarm hot water to a large number of patients suffering from peripheral vascular occlusive disease in Europe and revealed promising results in intermittent claudication [1], atherosclerosis [2], and chronic active hepatitis B [3].

It was reported to have both anti- and pro-oxidative properties *in vitro *[4]. In mouse macrophages PADMA 28 was found to inhibit inducible nitric oxide synthesis (iNOS) by decreasing iNOS mRNA and iNOS protein levels [5]. Recently, Neurauter et al. demonstrated that PADMA 28 modulates interferon γ-induced tryptophan degradation and neopterin production in human peripheral blood mononuclear cells (PBMC) [6]. Others have reported on its anti-inflammatory and anti-atherogenic properties [7].

It is generally accepted that improper functioning of cellular suicide or apoptosis is one of the master switches in tumourigenesis [8-10]. Accumulation of cells in subG1 phase, degradation of poly (ADP-ribose) polymerase (PARP) and nuclear body formation are hallmarks of apoptosis. In this study, using CEM-C7H2 leukaemia cells, we investigated whether and in what way PADMA 28 influences apoptosis.

Bcl-2 is the central gene family member involved in balancing between cell death and cell survival [11]. In the present study, the influence of PADMA 28 on Bcl-2 signalling in CEM-C7H2 cells deficient in functional p53 and p16 signalling was studied in a CEM sub-line constructed with the *Bujard system *in which the Bcl-2 gene is induced upon withdrawal of tetracycline [12].

## Methods

### Test substance

PADMA 28 was provided in powder form as an active substance mixture without excipients by PADMA Inc. Schwerzenbach, Switzerland. The herbal mixture is a fine brown dusty powder with a characteristic pungent smell. It is a mixture of a variety of different herbs (403 mg of active ingredients): *Aegle marmelos *(Bengal Quince) fruit (20 mg); *Pimenta dioica *(Allspice) fruit (25 mg); *Aquilegia vulgaris *(Columbine) aerial part (15 mg); *Calendula officinalis *(Marigold) flower (5 mg); *Elettaria cardamomum *(Cardamom) fruit (30 mg); *Syzygium aromaticum *(Clove) flower bud (12 mg); *Saussurea lappa *(Saussuria) root (40 mg); *Hedychium spicatum *(Hedychium) rhizome (10 mg); *Lactuca sativa *(Lettuce) leaf (6 mg); *Cetraria islandica *(Iceland moss) thallus (40 mg); *Glycyrrhiza glabra *(Licorice) root (15 mg); *Azadirachta indica *(Margosa) fruit (35 mg); *Terminalia chebula *(Myrobalan) fruit (30 mg); *Plantago lanceolata *(Ribwort) aerial part (15 mg); *Polygonum aviculare *(Knot-grass) aerial part (15 mg); *Potentilla aurea *(Golden Cinquefoil) aerial part (15 mg); *Pterocarpus santalinus *(Red Sandalwood) wood (30 mg); *Sida cordifolia *(Heart-leaved Sida) aerial part (10 mg); *Valeriana officinalis *(Valerian) root (10 mg); and *Aconitum napellus *(Monkshood) tuber (1 mg). Also present are 2 non-herbal components: *Dextrocamphora *(natural camphor) (4 mg) and *Calcii sulphas pulv*.(Gipsum; 20 mg).

In parallel, a derivative of this mixture, PADMA Basic, was also studied and its effects compared to PADMA 28. In view of different pharmaceutical standards in Austria and United States, *Aconitum napellus *is omitted in PADMA Basic.

Since the data on PADMA 28 were fully concordant with those on PADMA Basic only figures showing PADMA 28 data are shown.

Individual constituents as well as the whole mixtures were inspected and manufactured according to good manufacturing practice (GMP). The presence of heavy metals, pesticides and organic phosphate was controlled by high pressure liquid chromatography (HPLC). Thin layer chromatography, using a TLC-scanner, was used to fingerprint each batch of every individual component (e.g. essential oils). Terpenes were monitored by gas chromatography and mass spectrometry. Batches were rejected when results of fingerprinting differed substantially from that of reference material.

### Cell lines, culture conditions and transient transfection procedures

CEM-C7H2, a glucocorticoid-sensitive sub-line of CCRF-CEM-C7 cells [13], was cultured in RPMI 1640 medium (PAA Laboratories, Linz, Austria) supplemented with 10% heat-inactivated fetal calf serum (Biochrom, Berlin, Germany), 100 U/ml penicillin, 100 μg/ml streptomycin (Bio-Whittaker, Walkersville, MD), and 2 mM L-glutamine (Serva, Heidelberg, Germany) at 37°C in a humidified environment of 95% air and 5% CO_2_. African green monkey kidney fibroblasts (COS-1, 10^6^/100-mm dish) cells were kept at logarithmic growth phase in Dulbecco's Modified Eagle Medium (DMEM) supplemented with 10% heat-inactivated fetal calf serum, and 2 mM L-glutamine in a humidified atmosphere containing 5% CO_2_.

Two h post-transfection, cells were washed with pre-warmed DMEM and kept in RPMI. 24 h before harvesting the cells PADMA 28 extracts or solvent controls were added.

Stock solutions of PADMA 28 and PADMA Basic were prepared in ethanol (EtOH 70%, v/v), methanol (MeOH), and dimethylsulfoxide (DMSO, 1:1 distilled water) and kept at -20°C. Powdered PADMA 28 (400 mg) was vigorously mixed in a total volume of 2 ml of solvent and shaken for 30 min at 180 rpm at 37°C. The mixture was centrifuged at 2,000 × g for 10 min and the supernatants were sterile-filtered (pore size 0.22 μm). Cells were treated with various concentrations of PADMA 28 or PADMA Basic (final concentration range 0.4 mg/ml (1:500) – 1.0 mg/ml (1:200) solvent).

In parallel experiments, effects of pure ethanol, methanol and DMSO (Merck, Darmstadt, Germany) in concentrations comparable to the highest concentration in diluted PADMA 28 extracts were investigated. Viability of cells after application was determined by the trypan blue exclusion method.

### Fluorescence Imaging

48 h after transfection, cover slips were washed twice with PBS (140 mM NaCl, 2.7 mM KCl, 4.6 mM Na_2_HPO_4 _× 12 H_2_O, 1.3 mM NaH_2_PO_4 _× H_2_O), and cells were fixed with a 4% solution of p-formaldehyde for 15 min and if necessary, the nuclei were stained by addition of 1 μg/ml DAPI (4',6'-diamidine-2'-phenylindole dihydrochloride, Roche Molecular Biochemicals) diluted in PBS for 10 min. Finally, the cover slips were washed three times with PBS and mounted on slides with Mowiol mounting solution (Calbiochem, Switzerland). Microscopy studies were performed by employing an Olympus BX50 fluorescence microscope and images were taken with the digital camera Microview TE/CCD1317-K/1 (Princeton Scientific Instruments, USA). Imaging analysis was performed by using the *Metamorph imaging software *(Universal Imaging Corporation).

### Flow Cytometry

Detection of apoptotic cells was by FACS analysis of nuclear propidium iodide stained CEM cells. Briefly, 5 × 10^5 ^cells were permeabilized and stained with 750 μl propidium iodide (50 μg/ml in 0.1% Triton X-100/0.1% sodium citrate) and subjected to apoptosis analysis in a *FACScan *(FL-2 channel, Becton Dickinson, San Jose, CA, USA; equipped with an argon laser). Based on propidium iodide staining, cells in the sub-G1 marker window were considered to be apoptotic. Along with nuclear propidium iodide fluorescence, light scattering was also measured. According to the light scattering analysis, apoptotic nuclei are recognized as being smaller (lower forward scatter values) and more granulated (higher sideward scatter values). Cell debris and small particles were excluded from the analysis by forward/sideward scatter criteria as described elsewhere [14].

### PARP cleavage

CEM-C7H2 cells were seeded in 100-mm diameter dishes. Logarithmically growing cells were washed once with ice-cold phosphate-buffered saline (PBS) and lysed in 0.5 ml of Triton X-100 lysis buffer (50 mM Tris/HCl, pH 7.3, 50 mM NaCl, 5 mM Na_2_HPO_4 _× 12 H_2_O, 5 mM NaF, 5 mM EDTA, 50 μg/ml of both aprotinin and leupeptin, and 2% Triton X-100) for 20 min on ice. Lysates were clarified by centrifugation at 10.000 × g for 10 min at 4°C after a subsequent sonification procedure. Protein concentrations were determined according to Bradford. After boiling the samples for 5 min, equal amounts of proteins were separated on a 10% SDS-polyacrylamide gel electrophoresis. Transfer to Immobilon-P PVDF membranes (Millipore, Vienna) was in 25 mM Tris/HCl, pH 7.5, 193 mM glycine, 20% (v/v) methanol, and 0.1% SDS at 300 mA constant for 1 h. After blotting, membranes were soaked for 10 min in methanol and washed three times in TBST (50 mM Tris/HCl, pH 7.5, 150 mM NaCl, and 0.5% (v/v) Tween 20). Membranes were blocked overnight with TBST containing 5% (w/v) skimmed milk powder at 4°C and then probed with mouse anti-PARP antibody (diluted at 1:200 in blocking solution, Calbiochem, catalogue number 30-100UG) for 1 h at room temperature. After washing in TBST (4 times for 15 min) the blots were incubated with horseradish peroxidase-conjugated anti-mouse IgG (1:2000 dilution in blocking solution) for 45 min at room temperature. Membranes were washed again in TBST (4 times, 15 min) and immuno-reactive bands were visualized by enhanced chemiluminiscence detection (ECL, Amersham, Vienna, Austria).

## Results

**Figure 1:**
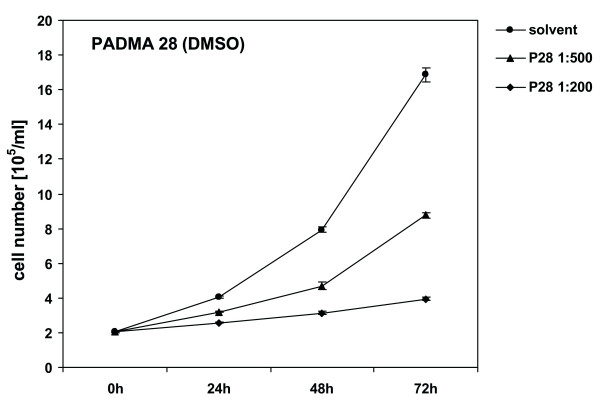
Influence of PADMA 28 on cell proliferation in CEM-C7H2 cells. After a growth period of 24 h, CEM-C7H2 cells were treated with different concentrations of PADMA 28 (0.4 mg/ml (1:500) and 1 mg/ml (1:200). After various application periods, cells were harvested, washed with ice-cold PBS and counted in a Coulter counter to determine cell proliferation rates. Cell dimension gates were set at 8,55 and 29,95 μm. Representative cell numbers are shown. No unspecific effects of the solvent controls were detectable during the incubation period in the cell culture. Data are expressed as cell numbers of untreated controls (± SEM, p < 0.01) of at least three independent experiments done in triplicate. For comparison of grouped data Student's t-test was applied. P-values below 0.05 were considered to indicate significant differences.

*Proliferation, inhibition and morphological alterations of CEM-C7H2 cells induced by PADMA 28*. CEM-C7H2 cells were treated with various concentrations of PADMA 28. Proliferation was determined by counting cell numbers in a Coulter counter. Figure 1 shows that application of various concentrations of PADMA 28 (0.4 mg/ml; 1.0 mg/ml) inhibited CEM cell proliferation in a concentration- and time-dependent manner. Adding PBS solved extracts at the same concentrations were less effective than alcoholic extracts, suggesting that pharmacologically active ingredients of PADMA 28 are extracted better by unpolar solvents. DMSO alone at the concentrations applied did not have any effect on cell proliferation.

**Figure 2:**
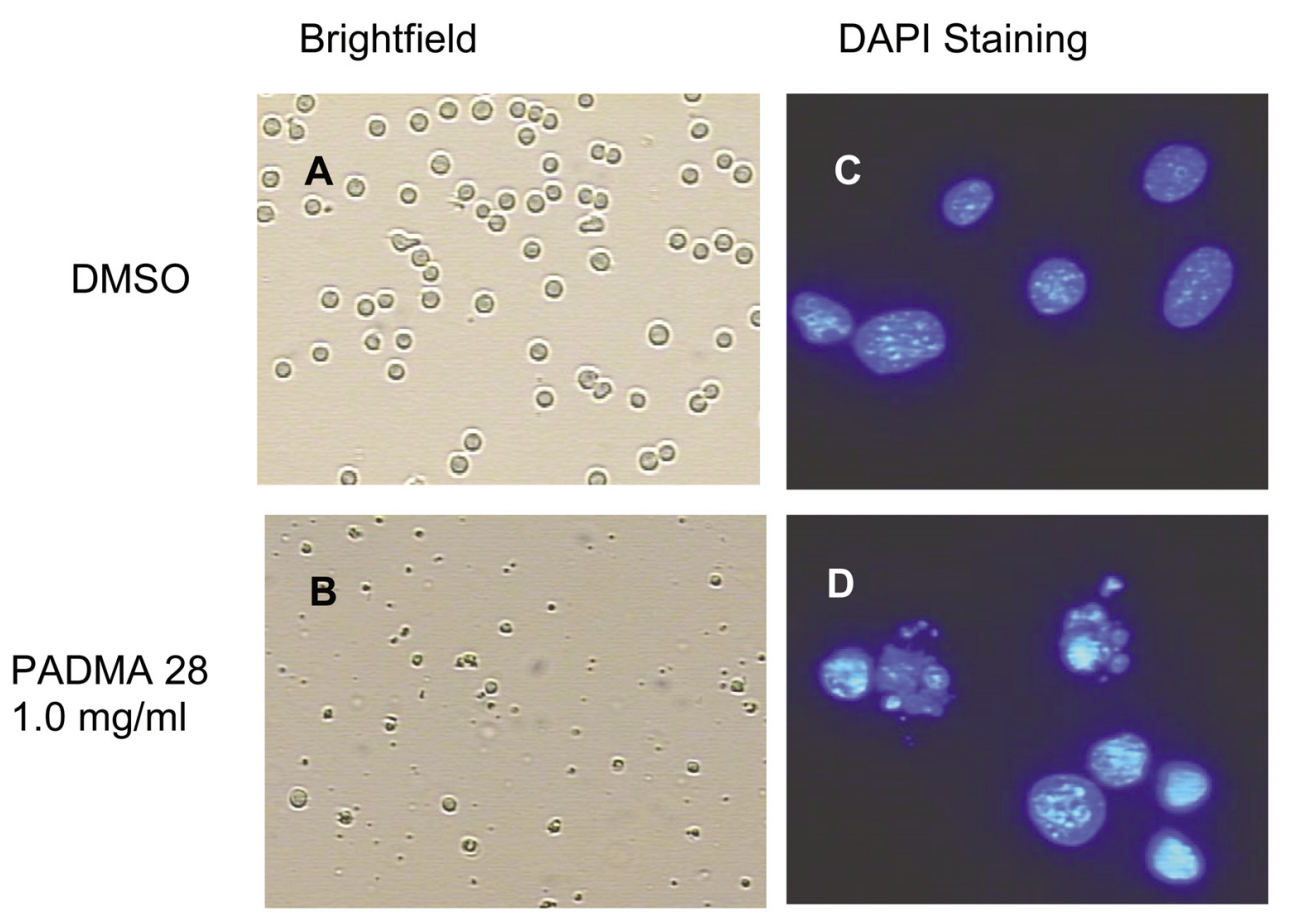
Morphological alterations of CEM-C7H2 cells after treatment with PADMA 28. CEM-C7H2 cells were either treated with solvent (DMSO) or 0.4 mg/ml of PADMA 28 for 48 h. Cells were stained with DAPI (right panels C/D; 100x) or left unstained (left panels A/B; 10x). Nuclear body formation was examined by fluorescence microscopy. Morphological alterations were analysed by employing transmitted light microscopy (Zeiss) of cells growing on glass cover slips.

In cultivated CEM cells treated with PADMA 28, morphological alterations of the nuclei characteristic for apoptosis were visible. This is shown in panel D of Figure 2.

### PADMA 28-induced subG1 transition

The G1 phase and the G1/S transition state of the cell cycle are critical points at which the cell assesses whether it should enter another full round of division, stop growing, or die.

**Figure 3:**
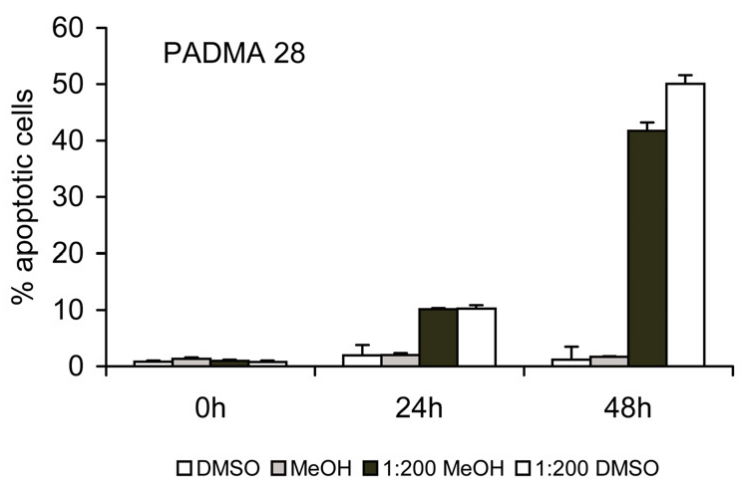
PADMA 28-induced subG1 transition. SubG1 transition was detected by employing FACS analysis as described under Methods. Representative subG1 populations calculated from FACS histograms are shown. Data are expressed as the percentage of subG1 cells calculated as the means (± SEM., n = 9, p < 0.01) of three independent experiments done in triplicate. As indicated, identical results were obtained with another solvent.

Transition of cell populations from G1/Go to subG1 phase is a frequent sign of apoptosis. As shown in Figure 3, application of 1 mg/ml PADMA 28 for 48 h resulted in accumulation of approximately 50% of cells in subG1 phase.

### PARP cleavage kinetic

**Figure 4:**
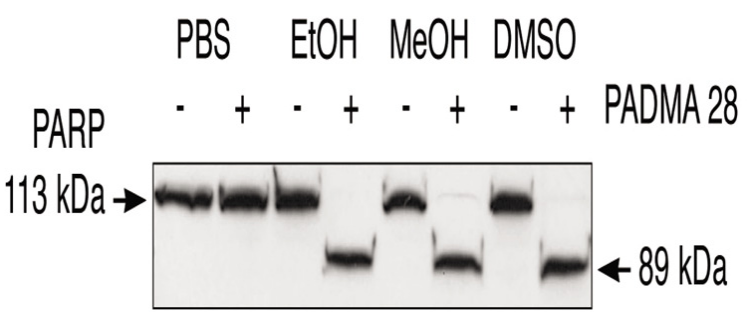
Kinetics of PARP cleavage. CEM-C7H2 cells were cultivated as described under Methods. Following treatment of cells with 0.1 mg/ml PADMA 28 for 24 h, PARP cleavage was detected by Western blotting. A representative cleavage pattern out of three independent experiments is shown. Upper bands show uncleaved PARP (113 kDa), lower bands correspond to a PARP cleavage product of 89 kDa. As shown for the growth inhibitory effect, PBS solutions were less effective than ethanolic or methanolic ones.

Activation of pre-existing pro-caspases takes place in a hierarchically ordered sequence starting with activation of initiator caspases and leading to activation of effector caspases such as caspase 3. Caspases 3 cleaves a number of proteins, a prominent substrate being poly (ADP-ribose) polymerase (PARP) [15]. PARP is a zinc-finger DNA-binding enzyme that detects DNA strand breaks generated by genotoxic agents such as oxygen radicals. PARP is activated at an intermediate stage of apoptosis and is inactivated by proteolytic cleavage at a late stage by casapase 3 and caspase 7. We raised the question whether treatment of CEM cells with PADMA 28 causes a caspase 3- or 7-mediated proteolytic cleavage of PARP. Figure 4 shows this was indeed the case. Treatment of CEM-C7H2 cells for 34 h caused a distinct proteolytic fragmentation of PARP, with accumulation of the 89 kDa fragment (lower bands) and concomitant disappearance of the full-size 113 kDa (upper bands) fraction (Figure 4, slot 4, 6, and 8). As was the case with growth inhibitory effect, PBS solutions of PADMA 28 were less effective than ethanolic, methanolic or DMSO extracts.

### PADMA 28 interferes with Bcl-2 function

**Figure 5:**
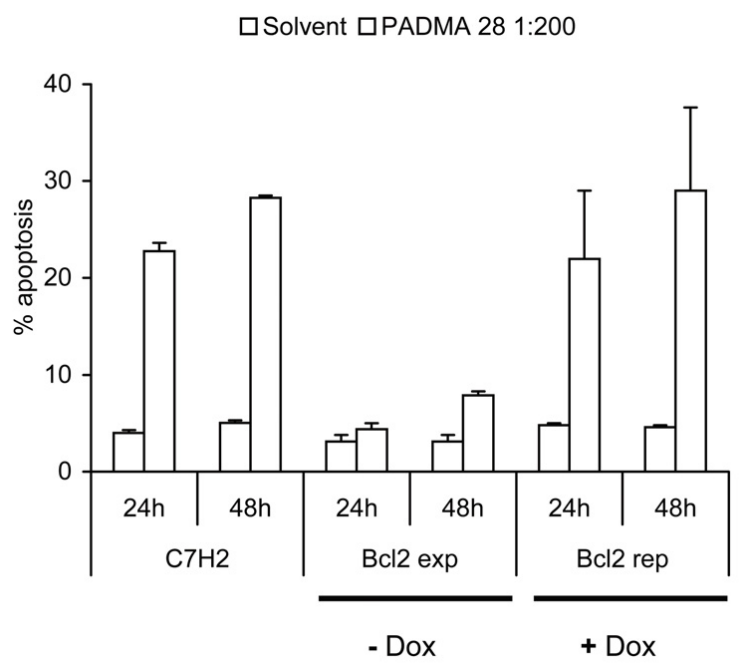
PADMA 28 interferes with Bcl-2 function. CEM-C7H2 as well as CEM-C7H2Bcl2-TET-off cells were cultivated in the presence (Bcl2 repressed) or absence (Bcl2 expressed) of 200 μg/ml tetracycline; 24 h after seeding, cells were treated with a solution of 0.4 mg/ml PADMA 28 (1:200 EtOH). After 24 and 48 h, cells were harvested and analysed by FACS as described under Methods. Data are expressed as percent of untreated solvent controls (± SEM, ± SEM, p < 0.03) of at least three independent experiments done in triplicate.

Whether a cell lives or dies is largely determined by the Bcl-2 function [11]. If PADMA 28 triggers apoptosis by interfering with pro-survival Bcl-2 signalling, it should be possible to diminish the pro-apoptotic signal by overexpression of Bcl-2. As shown in Figures 5/6, PADMA 28-mediated induction of apoptosis was diminished (Figure 5, bar 6 and 8) by transient overexpression of Bcl-2 in a CEM cell sub-line in which expression of the corresponding trans-gene was induced by withdrawal of doxocycline (Bujard system, CEM C7H2 Bcl-2 TET off).

**Figure 6:**
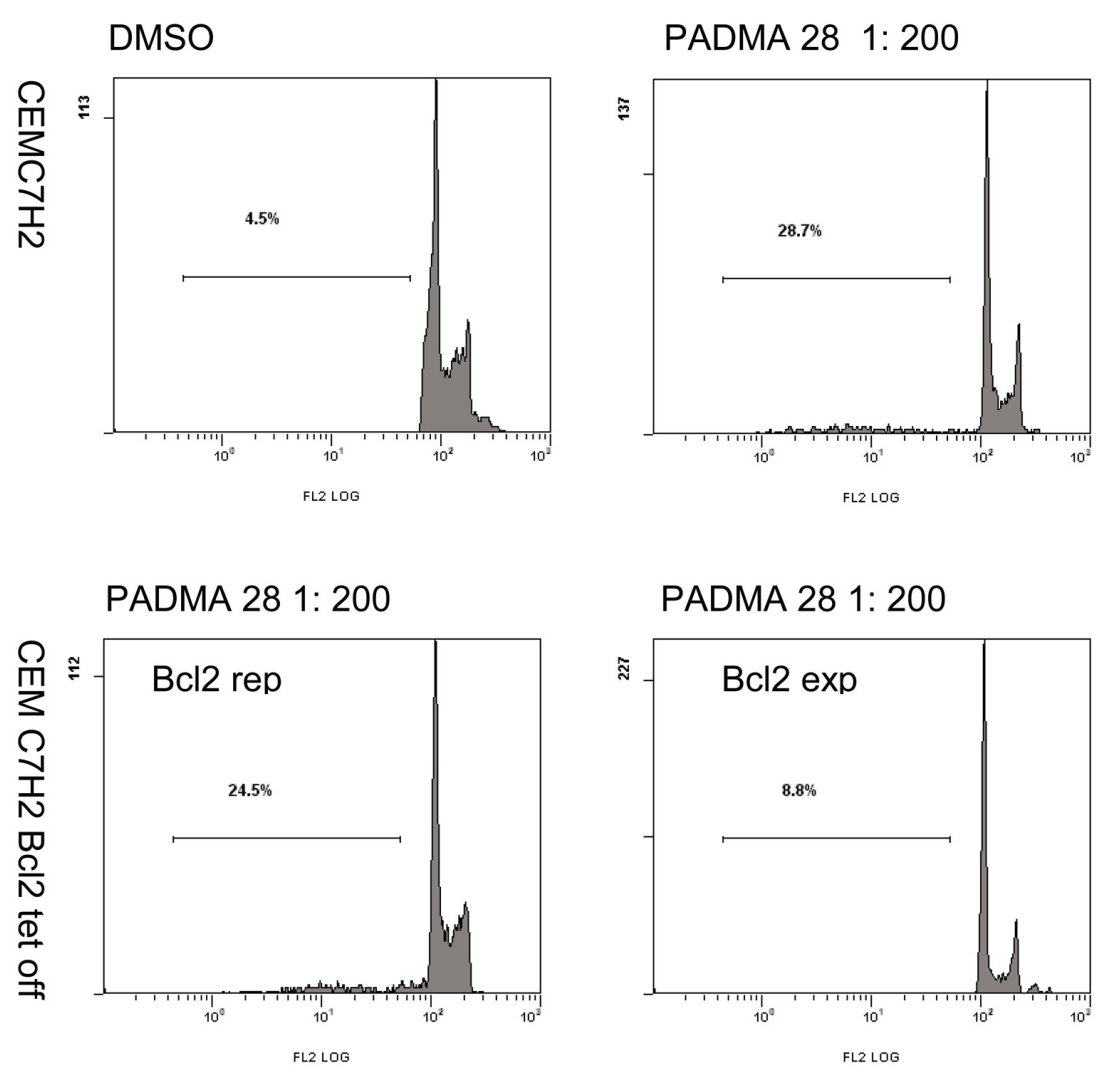
Corresponding FACS blots of figure 5.

Bcl-2 expression is controlled, to some extent, by liberation of the Th1-type cytokine IFN-γ [16]. Hence we would like to suggest that PADMA 28 may modulate apoptogenic pathways controlled by interferon-γ.

In this context it should be mentioned that PADMA 28 has been reported to modulate interferon-γ-induced tryptophan degradation and neopterin production in human peripheral blood mononuclear cells (PBMC) from healthy donors [6].

## Discussion

Here we show that PADMA 28 added to T cell-derived lymphatic leukaemia cells in culture has a pronounced concentration- and time-dependent anti-proliferative effect. The inhibition of cell proliferation is accompanied by nuclear body formation a morphological signs of apoptosis. Furthermore hallmarks of apoptosis, such as the accumulation of cells in subG1 phase, and fragmentation of poly (ADP-ribose) polymerase (PARP) could be detected.

A growing body of evidence demonstrates that cell survival and cell death are largely determined by the function of Bcl-2 family members. Here we show that induction of apoptosis by PADMA 28 can be effectively counteracted by overexpression of the pro-survival protein Bcl-2. Based on our data we suggest that PADMA 28 may act in a pro-apoptotic fashion by interfering with Bcl-2 signalling.

Bcl-2 expression is, partially, controlled by interferon-γ [16,17]. An influence of PADMA 28 on interferon-γ-mediated signals was recently demonstrated by Neurauter et al. [6] in peripheral blood mononuclear cells (PBMCs). They found that PADMA 28 has a pronounced effect on interferon-γ-stimulated neopterin production and tryptophan degradation in stimulated PBMC [6]. This observation appears to be of particular importance since cytokine-induced tryptophan degradation by indoleamine (2,3)-dioxygenase (IDO) is a potent mechanism to induce apoptosis and T-cell tolerance via deprivation of the essential amino acid tryptophan and by the pro-apoptotic activity of several tryptophan catabolites formed upon this enzyme reaction [18]. Moreover, evidence is accumulating that activation of IDO may represent an immune escape mechanism of tumour cells [19].

Assuming that stimulation of PBMC by mitogens elicits oxidative stress in culture, it seems reasonable to suggest that the above-mentioned effect of PADMA 28 is due to its ability to function as an antioxidant. This assumption is in agreement with findings that PADMA 28 decreases iNOS mRNA levels of mouse macrophages [5], a fundamental process that might contribute to the anti-inflammatory activities demonstrated by others [2-7].

Although PADMA 28 has been demonstrated to inhibit cell proliferation and cause apoptosis under cell culture conditions, one major question is left unanswered: is there any therapeutic window for using a multifactorial herbal remedy, such as PADMA 28, as a supplement in the treatment of leukaemia patients?

Chemotherapeutic agents are most effective when administered in combination (e.g. CHOP, chemotherapeutic multifactorial regimen comprising of doxorubicin, cyclophosphamide, vincristin and prednisone). Traditional Chinese or Tibetan medicine uses mixtures of naturally occurring herbs and herbal extracts for cancer therapy. Many of the commonly used herbal remedies that have been tested have yielded activities that disappeared when the extracts were fractionated into individual chemical components. We agree with Curtis T. Keith et al. (Nature Reviews, Drug Discovery, 2005) [20] that a focus on multicomponent therapeutics might lead to new insights in drug discovery and that multifactorial therapeutics are necessary for the treatment of multifactorial diseases. In agreement with this concept, PADMA 28, a multifactorial herbal formula, might be similarly effective as a chemotherapeutic agent administered as a supplement, and/or be able to minimize the toxic side effects of such a regimen.

It is not clear to what extent one can extrapolate results from our cell culture experiments to possible effects in humans. Not all of the compounds can be efficiently absorbed, and a gradient exists between the concentrations present in the extracts and the ones established in the blood. Nevertheless, one can assume the presence of all ingredients in the gastrointestinal tract at reasonable concentrations, which makes anti-inflammatory and pro-apoptotic potency more likely. This assumption is further reinforced by the fact that PADMA 28 is successfully used to treat intermittent claudication, atherosclerosis, scleroderma, multiple sclerosis, and chronic hepatitis.

Clinical trials are required for determining whether PADMA 28 can be effectively used in the treatment of cancers. However, biochemical studies might help bridge the gap between conventional and alternative tumor therapy strategies. Additional studies focusing on the biological significance of PADMA 28-enhanced apoptosis in various cell systems will greatly extend our understanding of the mode of action of this remedy. In order to clarify the biological function of PADMA 28 in molecular fashion, gene expression profiling experiments by employing Affymetrix technology are currently under way.
